# Protection of *Inonotus hispidus* (Bull.) P. Karst. against Chronic Alcohol-Induced Liver Injury in Mice via Its Relieving Inflammation Response

**DOI:** 10.3390/nu15163530

**Published:** 2023-08-10

**Authors:** Xinghui Jin, Zhige Li, Yongfeng Zhang, Yanfeng Zhu, Ling Su, Jiyu Song, Jie Hao, Di Wang

**Affiliations:** 1 School of Life Sciences, Jilin University, Changchun 130012, China; jinxh21@mails.jlu.edu.cn (X.J.); lizg21@mails.jlu.edu.cn (Z.L.); yongfeng20@mails.jlu.edu.cn (Y.Z.); zhuyf21@mails.jlu.edu.cn (Y.Z.); 2 Engineering Research Center of Chinese Ministry of Education for Edible and Medicinal Fungi, Jilin Agricultural University, Changchun 130118, China; suling0648@jlau.edu.com; 3 Department of Orthodontics, Hospital of Stomatology, Jilin University, Changchun 130021, China; songjy20@mails.jlu.edu.cn

**Keywords:** *Inonotus hispidus*, hepatoprotection, chronic alcohol damage, inflammation

## Abstract

Alcoholic liver disease (ALD) can be induced by excessive alcohol consumption, and has a worldwide age-standardized incidence rate (ASIR) of approximately 5.243%. *Inonotus hispidus* (Bull.) P. Karst. (IH) is a mushroom with pharmacological effects. In ALD mice, the hepatoprotective effects of IH were investigated. IH strongly ameliorated alcohol-induced pathological changes in the liver, including liver structures and its function-related indices. Intestinal microbiota and serum metabolomics analysis showed that IH altered the associated anti-inflammatory microbiota and metabolites. According to results obtained from Western blot, immunohistochemistry (IHC), and enzyme-linked immunosorbent assay (ELISA), IH downregulated the levels of pro-inflammation factors interleukin (IL)-1β, IL-6 and tumor necrosis factor-α (TNF-α), enhanced the expressions of peroxisome proliferator-activated receptor alpha (PPARα) and 15-hydroxprostaglandin dehydrogenase (15-PGDH), and inhibited the phosphorylated activation of Janus kinase (JAK) 1 and signal transducer and activator of transcription (STAT) 3, confirming the hepatoprotection of IH against alcohol damage via anti-inflammation. This study provides the experimental evidence for the hepatoprotective effects of IH in chronic ALD.

## 1. Introduction

Alcoholic liver disease (ALD) includes steatosis, alcoholic hepatitis, liver fibrosis and cirrhosis [1]. According to a systematic analysis of the global burden of ALD [2], the age-standardized incidence rate (ASIR) for ALD was approximately 5.243%, while the age-standardized death rate (ASDR) was around 4.48% [2]. Drugs used for ALD therapy include glucocorticoids, pentoxifylline and metadoxine, which have various side effects [3]. Silymarin (Sil), one of the representative agents, can cause gastrointestinal adverse events after chronic administration [1,4].

Based on previous research, the intestinal microbiota and associated metabolites can influence the process of ALD [5]. Changes in the intestinal microbiota are evident in patients with ALD and are associated with liver inflammation [6]. Liver inflammation is an important phenomenon related to ALD [7]. Signal transducer and activator of transcription (STAT) 3 is a transcription factor activated by Janus kinase (JAK) 1 and is associated with liver inflammation [8]. Excessive alcohol consumption induced the increase in interleukin (IL)-6, IL-1β, and tumor necrosis factor-α (TNF-α), which belong to the pro-inflammatory factors that strongly promote the development of ALD [9]. ALD, accompanied by lipid metabolic disorders, induces inflammation [10]. Peroxisome proliferator-activated receptor alpha (PPARα), an important regulator of fat transport and oxidation, is inactivated by acetaldehyde, which is oxidized from alcohol and is a hub for inflammation and lipid metabolism [11]. 

Mushrooms are natural products with lots of biological properties, including relief from inflammation and even liver diseases [12]. *Inonotus hispidus* (Bull.) P. Karst. (IH), a medicinal mushroom belonging to the *Inonotus* genus of the *Hymenochaetaceae* family, is distributed in many places in China [13]. Current studies on IH have focused on the extraction process [14], pharmacological activity [15] and compositional analysis [16]. IH improves hyperuricemia and gouty arthritis through anti-inflammatory effects [17]. IH spore powder affects the intestinal microbiota and serum metabolites in B6/JGpt-Apc*^em1Cin(min)^*/Gpt (Apc*^Min/+^*) mice, a model of colorectal cancer, which in turn affects the JAK/STAT signaling and regulates the abundance of CD8^+^ T cells to suppress the development of colorectal cancer (CRC) [18]. IH extracellular exopolysaccharide ameliorates acute ALD by mediating the Nrf2 signaling pathway and upregulating the levels of antioxidant-related factors [13]. However, the effects of IH on chronic ALD have yet to be systematically reported.

In our study, IH showed hepatoprotective activity in chronic ALD mice, partly reversed the effects of alcohol on intestinal microbiota and serum metabolites, and downregulated the levels of inflammatory cytokines, providing experimental evidence for the application of IH on chronic ALD administration.

## 2. Materials and Methods

### 2.1. IH Preparation

The fruiting body of IH (the voucher specimens were deposited at the Engineering Research Center of Edible and Medicinal Fungi, Ministry of Education, Jilin Agricultural University in Changchun City, Jilin Province, China; the voucher number for the specimens is CCMJ2775) was cultured by Jilin Agriculture University in Yanji City, Jilin Province, China and collected, and identified by Prof. Yu Li from Jilin Agriculture University. The fruiting body of IH was cleaned, dried, and crushed into coarse particles which were subsequently milled into ultrafine powder and stored under dry conditions.

### 2.2. Animal Experimental Procedures

**Figure 1:**
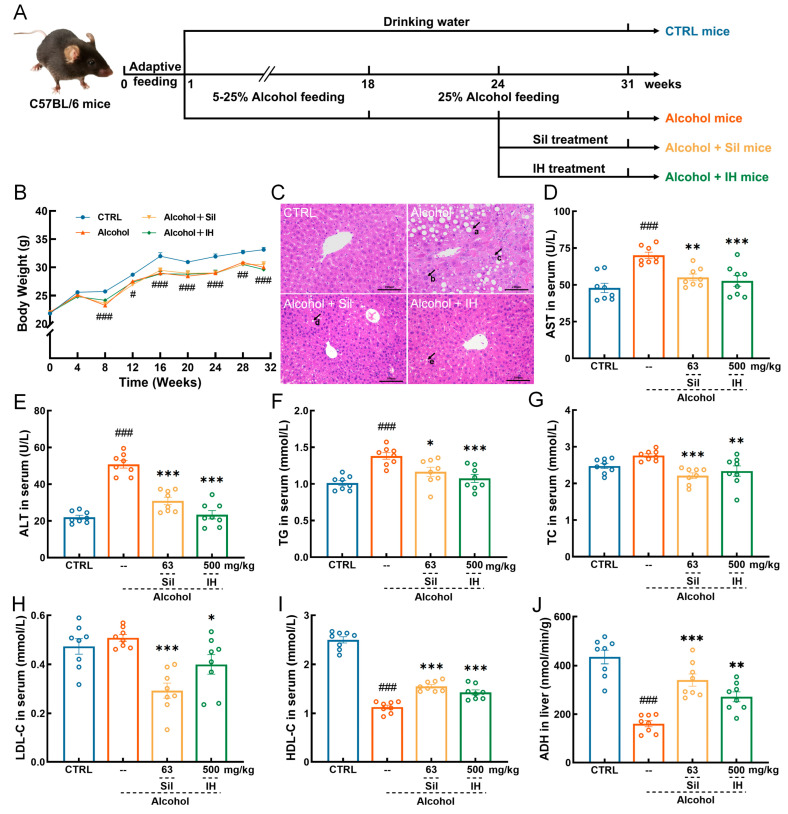
*Inonotus hispidus* (Bull.) P. Karst. (IH) alleviated chronic liver damage caused by alcohol. (**A**) Animal model establishment and agent administration process. (**B**) Body weight of the mice was measured (*n* = 12). (**C**) Hematoxylin and Eosin (H&E) staining was used to analyze the histopathological changes of the liver in mice with chronic alcoholic liver injury (*n* = 3) (200×, scale bar: 100 μm) (a. large number of fatty vacuoles, b. hepatocellular swelling, c. inflammatory cell infiltrate, d, e. few fatty vacuoles). Liver-related indicators (**D**) aspartate aminotransferase (AST), (**E**) alanine aminotransferase (ALT), (**F**) triglyceride (TG), (**G**) total cholesterol (TC), (**H**) low-density lipoprotein cholesterol (LDL-C), (**I**) high-density lipoprotein cholesterol (HDL-C), (**J**) alcohol dehydrogenase (ADH) were analyzed to verify the alleviating effect of IH on liver injury (*n* = 8). The data are shown as the mean ± S.E.M. # *p* < 0.05, ## *p* < 0.01, ### *p* < 0.001 vs. control (CTRL) mice; * *p* < 0.05, ** *p* < 0.01, *** *p* < 0.001 vs. Alcohol mice.

Forty-eight male C57BL/6 mice (7–8 weeks, 20–24 g), were purchased from Liaoning Changsheng Biotechnology Co., Ltd. (Benxi, China) [SCXK (Liao) 2020-0001] and housed in ventilated cages under standard conditions: ambient temperature of 22–24 °C, relative humidity of 45–55%, a light/dark cycle of 12 h (lights on 7:00 a.m.–7:00 p.m.). All the mice were provided with ad libitum access to food and water [17]. As described in our previous study [19] (Figure 1A), after a week of acclimation, the mice were randomly divided into control (CTRL) mice (*n* = 12) and chronic ALD mice (*n* = 36). During the 31-week experimental period, the CTRL mice received drinking water. In contrast, the chronic ALD mice received 5% (*v*/*v*) alcohol in drinking water for one week, which was then gradually increased 5% alcohol per week, until the alcohol concentration in the drinking water reached 25%, and this lasted until the end of the experiment. Starting from the 18th week, the chronic ALD mice were intragastrically (i.g.) administered with 10 mL/kg of 25% alcohol additionally, and the CTRL mice received 10 mL/kg of drinking water (i.g.) daily for six weeks. From the 24th week, the chronic ALD mice were randomly divided into three groups (*n* = 12/group) [19]. In combination with receiving alcohol, the mice received 10 mL/kg normal saline orally (Alcohol mice), 63 mg/kg Sil (63 mg Sil was dissolved in 10 mL normal saline) (Alcohol + Sil mice), and 500 mg/kg IH (500 mg IH ultrafine powder was dissolved in 10 mL normal saline to form a suspension of IH that can be easily administered orally) (Alcohol + IH mice), daily, for eight weeks. During the experiment, the body weight of the mice was measured weekly. After the last administration, the mice were fasted for eight hours. Peripheral blood samples of mice were collected from the tail vein using a medical syringe, then mice were euthanized by CO_2_ inhalation. The liver, spleen, kidney, heart, lung, muscle, brain, and fecal samples from the cecum were rapidly removed. Part of the tissues and samples was flash-frozen and stored at −80 °C until analysis, and the remaining tissues were fixed in 4% paraformaldehyde. This study was approved by the Institutional Animal Ethics Committee of Jilin University (SY202112002). 

### 2.3. Hematoxylin and Eosin (H&E) Staining

The fixed organ tissues (liver, spleen, kidney, heart, lung, muscle, and brain) were dehydrated with graded concentrations of ethanol, embedded in paraffin, sliced into approximately 5 μm sections, stained with H&E and observed using a light microscope, as described in our previous study [20].

### 2.4. Biochemical Detection

Peripheral blood obtained from the mice was incubated at room temperature for 30 min and centrifuged twice at 825× *g* for 10 min to obtain serum. Serum concentrations of liver function-related factors were detected using biochemistry kits, following the instructions provided by the manufacturer. Liver tissues (30 mg) were homogenized thoroughly using a high-throughput tissue grinder with 300 μL phosphate buffer saline (PBS). After centrifuging twice at 825× *g* for 10 min, the protein concentration of the samples was detected using a bicinchoninic acid assay (BCA) kit, and the IL-1β, IL-6, and TNF-α in the supernatant of the liver samples were detected using an enzyme-linked immunosorbent assay (ELISA) kit. Alcohol dehydrogenase (ADH) was detected using the biochemical reaction method. ADH can catalyze the formation of a reactant with a maximum absorption peak at 450 nm from ethanol, and the activity of ADH was calculated by detecting the rate of increase of the substance at 450 nm. The details of all kits used for detection are listed in Supplementary Table S1.

### 2.5. Intestinal Microbiota Analysis

Bacterial DNA was extracted from fecal samples collected from the cecum using an OMEGA Soil DNA Kit. After PCR amplification, 16s rRNA was sequenced using a NovaSeq 6000 SP Reagent Kit on an Illumina NovaSeq platform (Personal Biotechnology, Shanghai, China), as previously described [21]. The 16s rRNA reads were denoised and analyzed using QIIME2 analysis software to obtain operable taxonomic units (OTUs) with a more than 97% similarity threshold. The α-diversity was calculated with Simpson, Chao1, Faith’s phylogenetic diversity (Faith’s PD), Good’s coverage, and Pielou’s evenness, and post hoc using the Kruskal–Wallis rank-sum test and Dunn’s test to determine the richness and diversity of the microbiota. β-diversity was analyzed using principal coordinate analysis (PCoA), based on the Bray–Curtis distance to evaluate the compositional diversity of microbial communities. Linear discriminant analysis (LDA) effect sizes were used to analyze the microbiota features among the experimental groups.

### 2.6. Non-Targeted Metabolomics Analysis

Non-targeted metabolomics analysis of mice serum was performed as previously described [18]. The raw data were analyzed using Personalbio Technology Co., Ltd. (Shanghai, China), Metabolites, with an adjusted *p*-value < 0.05, and variable important in projection (VIP) > 1 were assigned as differentially expressed.

### 2.7. Western Blot Analysis

Liver samples from each group were homogenized and lysed thoroughly using a radioimmunoprecipitation assay buffer containing 1% protease and phosphatase inhibitors to obtain whole-protein samples. The quantified proteins were separated by sodium dodecyl sulfate-polyacrylamide gel electrophoresis, transferred to polyvinylidene difluoride membranes, blocked with a rapid closure solution, and sequentially incubated with primary and secondary antibodies to obtain protein bands, as previously described [20]. Protein bands were visualized using an ultra-high-sensitivity enhanced chemiluminescence kit. Information on all antibodies is shown in Table S2.

### 2.8. Immunohistochemistry (IHC) Analysis

Paraffin sections of liver were deparaffinized with a deparaffinizing solution (G1128; Servicebio, Wuhan, China), rehydrated with different concentrations of alcohol, and washed in PBS (G0002; Servicebio, Wuhan, China) three times. The sections were incubated in 3% H_2_O_2_ solution at room temperature for 25 min in dark conditions to block endogenous peroxidase, blocked with a rapid closure solution at room temperature for 30 min, and finally sequentially incubated with primary antibodies at 4 °C overnight and secondary antibodies at room temperature for 50 min. Sections were developed using diaminobenzidine color-developing solution (G1212; Servicebio, Wuhan, China) and re-stained with hematoxylin (G1004; Servicebio, Wuhan, China). Sections were observed and photographed using a light microscope (Nikon E100; Nikon Corporation, Tokyo, Japan). Information on all the antibodies is shown in Table S2.

### 2.9. Statistical Analysis

All values are expressed as the mean ± S.E.M. One-way analysis of variance (ANOVA) and post hoc multiple comparisons (Dunn’s test) were performed using DSS 25.0 (IBM Corporation, Armonk, NY, USA). *p* < 0.05 was considered to be statistically significant.

## 3. Results

### 3.1. IH Protected against Chronic Alcohol Injury in Mice

Compared to CTRL mice, alcohol consumption inhibited weight gain (*p* < 0.05, Figure 1B). In contrast, Sil and IH failed to reverse this phenomenon (Figure 1B). In chronic ALD mice, IH suppressed the indices of the heart (*p* < 0.01), lung (*p* < 0.01), and kidney (*p* < 0.001), but failed to influence the indices of the liver, spleen and thymus (Table S3). No significant histopathological changes were observed in the spleen, kidney, heart, lung, muscle, or brain of mice in any group (Figure S1). Compared to CTRL mice, chronic alcohol consumption caused hepatocyte enlargement, congestion, a disorder of the hepatic sinusoid, hepatocyte cords stenosis, many fat vacuoles of different sizes, a reduction in hepatocyte nuclei, and inflammatory cell infiltration in the liver, all of which were significantly reversed by Sil and IH treatments (Figure 1C). In long-term alcohol consumption mice, IH downregulated the levels of aspartate aminotransferase (AST) (*p* < 0.001, Figure 1D), alanine aminotransferase (ALT) (*p* < 0.001, Figure 1E), triglyceride (TG) (*p* < 0.001, Figure 1F), total cholesterol (TC) (*p* < 0.01, Figure 1G) and low-density lipoprotein cholesterol (LDL-C) (*p* < 0.05, Figure 1H), and enhanced the level of high-density lipoprotein cholesterol (HDL-C) (*p* < 0.001, Figure 1I) in serum. IH increased the level of ADH (*p* < 0.01, Figure 1J) in the liver of chronic ALD mice. Similar effects on these factors were noted in Alcohol + Sil mice (*p* < 0.05, Figure 1D–J).

### 3.2. IH Regulated the Intestinal Microbiota in Chronic ALD Mice 

**Figure 2:**
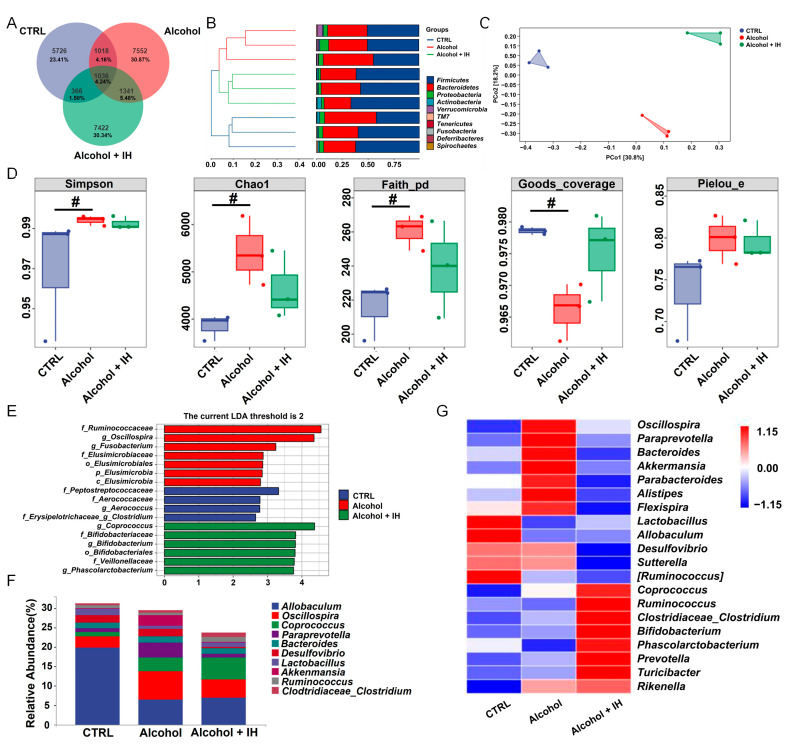
IH regulated intestinal microbiota in chronic alcoholic liver disease (ALD) mice (*n* = 3). (**A**) Venn diagram representation of shared/ unique operable taxonomic units (OTUs) in the intestinal microbiota of CTRL, Alcohol and Alcohol + IH mice. (**B**) Based on the Bray–Curtis hierarchical clustering analysis in the β-diversity clustering analysis, the similarity among the three groups was presented as a hierarchical tree. (**C**) Principal coordinate analysis (PCoA) of Bray–Curtis distance derived from β-diversity analysis was used to characterize the community differences between the three groups. (**D**) α-diversity indices of three different groups. # *p* < 0.05 vs. CTRL mice. (**E**) Histograms of the distribution of the linear discriminant analysis (LDA) values of significantly different species based on the results of linear discriminant analysis of effect size (LEfSe) analysis, also showing the significantly enriched species within each group. (**F**) Bacterial taxonomic profiling of intestinal microbiota at the genus level (top 10). (**G**) The heat map shows significant changes in the 20 genera with the highest abundance.

According to Venn diagram analysis, 24,461 OTUs were identified among the three groups, of which 5726 (23.41%), 7552 (30.87%) and 7422 (30.34%) specific OTUs were identified in the CTRL, Alcohol, and Alcohol + IH mice, respectively. A total of 1036 (4.24%) common OTUs were observed among the three groups (Figure 2A). Hierarchical clustering analysis revealed that *Firmicutes* and *Bacteroidetes* were the main bacterial phyla in all three groups (Figure 2B). For β-diversity analysis, the PCoA plot revealed a clear separation among the three groups, which indicated that IH treatment had different effects on the overall structure of the intestinal microbiota in mice with chronic alcohol consumption (Figure 2C). The α-diversity analysis is used to analyze the species diversity of the intestinal microbiota [22]. Compared to the CTRL mice, alcohol consumption significantly increased the diversity, richness, and evolutionary-based diversity, and decreased the coverage in the intestinal microbial community. These metrics were respectively expressed as the following indices: Simpson (*p* < 0.05), Chao1 (*p* < 0.05), Faith’s PD indices (*p* < 0.05) and Good’s coverage indices (*p* < 0.05, Figure 2D). Linear discriminant analysis of effect size (LEfSe) showed that IH significantly increased the abundances of genus *Coprococcus*, family *Bifidobacteriaceae*, genus *Bifidobacterium*, order *Bifidobacteriales*, family *Veillonellaceae* and genus *Phascolarctobacteruim* in chronic ALD mice (Figure 2E). The 10 most abundant genera and the 20 genera with the most significant differences were selected to analyze changes in the intestinal microbiota after alcohol accumulation and IH administration. In chronic ALD mice, IH increased the abundance of the *Phascolarctobacterium*, *Lactobacillus*, *Allobaculum*, and *Ruminococcus* and decreased the abundance of *Parabacteroides*, *Paraprevotella* and *Alistipes* (Figure 2F,G and Table S4).

### 3.3. IH Altered n-3 Polyunsaturated Fatty Acids (n-3 PUFAs) among Serum Metabolites in Chronic ALD Mice

**Figure 3:**
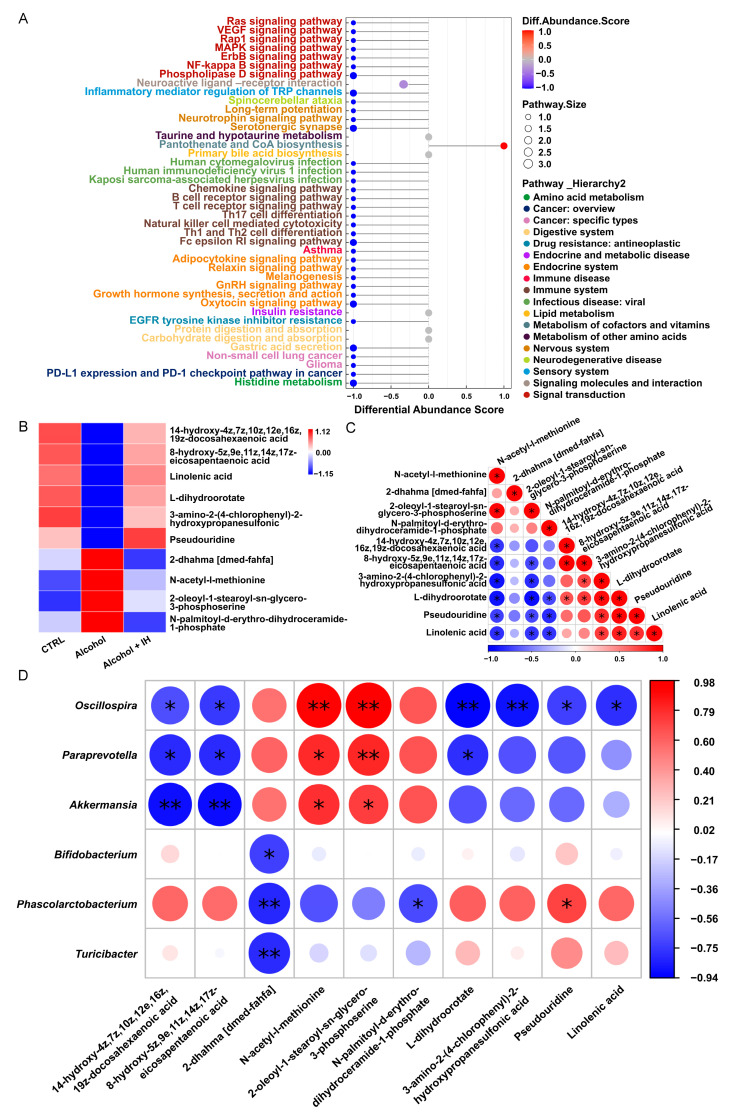
IH regulated serum metabolites in chronic ALD mice (*n* = 3). (**A**) Differential abundance scores of enriched metabolic pathways among the groups. A score of 1 indicates that all identified metabolites are upregulated, and −1 indicates that all identified metabolites in the pathway are downregulated. (**B**) The heat map of different serum metabolites. (**C**) The correlation analysis of altered metabolites in serum. * *p* < 0.05. (**D**) The correlation analysis of intestinal microbiota with differential metabolites. * *p* < 0.05, ** *p* < 0.01. Color keys represents the correlation coefficients.

A total of 1365 metabolites in the serum were measured from different groups of mice, more than half of which belong to lipids and lipid-like molecules (31.795%), as well as organic acids and derivatives (19.927%) (Figure S2). Functional analysis of metabolites was conducted using the Kyoto Encyclopedia of Genes and Genomes. The differential abundance score showed that the immune system and signal transduction occupied one third (14 of 42), both being associated with inflammation (Figure 3A). Based on the differential metabolites of the CTRL vs. Alcohol and Alcohol + IH vs. Alcohol mice, we screened the repetitive metabolites (Table S5), which were analyzed and were shown as a heat map and a correlation coefficient map. Ten metabolites were significantly altered in alcohol-treated mice, which IH reversed. We focused on Linolenic acid (ALA), 14-hydroxy-4z,7z,10z,12e,16z,19z-docosahexaenoic acid (14-HDoHE) and 8-hydroxy-5z,9e,11z,14z,17z-eicosapentaenoic acid (8-HEPE) from the 10 metabolites, all of which were related to *n*-3 PUFAs (Figure 3B,C). 14-HDoHE is a substrate for 15-hydroxprostaglandin dehydrogenase (15-PGDH), resulting in the formation of its electrophilic metabolite, 14-oxoDHA, which contributes to the anti-inflammatory signaling [23]. 8-HEPE induces the PPARα activation to regulate lipid metabolism and inhibits the inflammation indirectly [24,25]. ALA also has anti-inflammation properties like other *n*-3 PUFAs [26,27]. Interestingly, three of the ten metabolites are related to *n*-3 PUFAs, connected to anti-inflammation, which were upregulated in Alcohol + IH mice. 14-HDoHE and 8-HEPE showed positive correlations with ALA. Correlation analysis was conducted between the filtered metabolites and the 20 most abundant intestinal microbiota (Figure 3D). 14-HDoHE and 8-HEPE showed positive correlation with *Phascolarctobacterium* and significantly negative correlation with *Oscillospira* (* *p* < 0.05, Figure 3D). ALA also showed significantly negative correlation with *Oscillospira*. 

### 3.4. IH Suppressed Inflammation in Chronic ALD Mice

**Figure 4:**
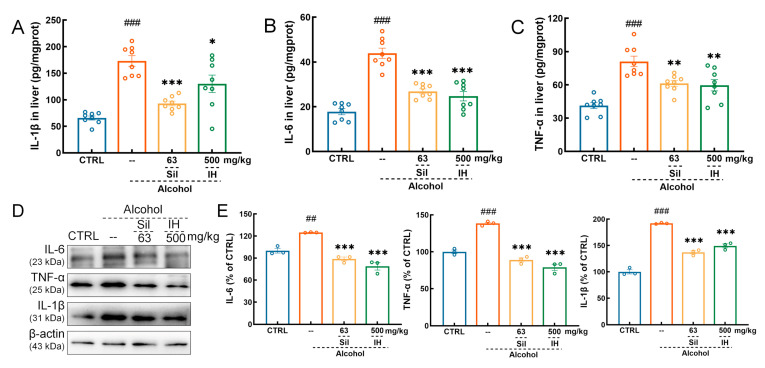
IH alleviated the inflammatory response in chronic ALD mice. IH decreased the expression levels of (**A**) interleukin (IL)-1β, (**B**) IL-6, and (**C**) tumor necrosis factor-α (TNF-α) in the liver, according to the enzyme-linked immunosorbent assay (ELISA) results (*n* = 8). (**D**) IH decreased the protein expression levels of IL-6, TNF-α and IL-1β in the liver of alcohol mice, according to the Western blot results. (**E**) Quantification of protein expression normalized to β-actin and expressed as the percentage of CTRL mice. The data are shown as the mean ± S.E.M. ## *p* < 0.01, ### *p* < 0.001 vs. CTRL mice; * *p* < 0.05, ** *p* < 0.01, *** *p* < 0.001 vs. Alcohol mice.

Therefore, relieving inflammation may play an important role in the IH-mediated protection of hepatocytes in chronic ALD mice. IH suppressed the levels of pro-inflammatory cytokines, including IL-1β (*p* < 0.05, Figure 4A), IL-6 (*p* < 0.001, Figure 4B), and TNF-α (*p* < 0.01, Figure 4C) in the liver. IH suppressed the levels of IL-6 (*p* < 0.001), TNF-α (*p* < 0.001) and IL-1β (*p* < 0.001) in the liver, which was further confirmed by Western blot (Figure 4D,E).

**Figure 5:**
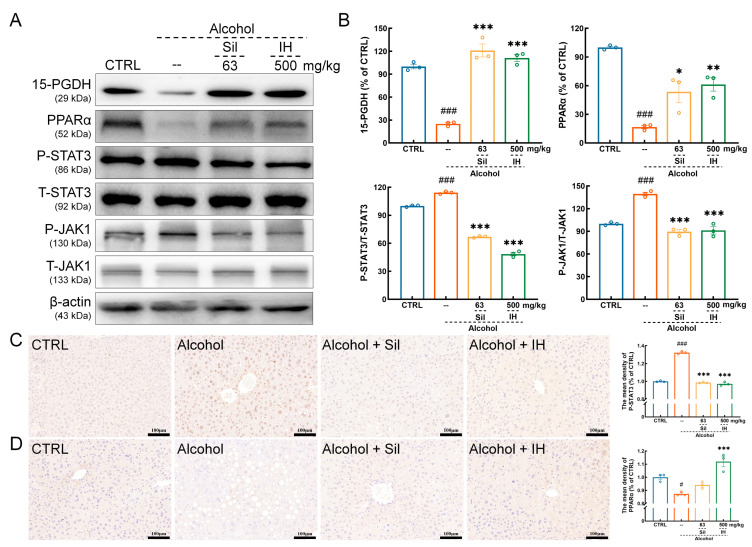
IH altered the inflammation-related factors in chronic ALD mice. (**A**) IH administration increased the levels of 15-hydroxprostaglandin dehydrogenase (15-PGDH) and peroxisome proliferator-activated receptor alpha (PPARα), and decreased the levels of phospho- (P-) Signal transducer and activator of transcription (STAT) 3 and P- Janus kinase (JAK) 1 in the liver of chronic ALD mice. (**B**) Quantification of protein expression normalized to β-actin or their related total proteins, and expressed as the percentage of CTRL mice. In immunohistochemical staining in mouse liver tissue, IH administration (**C**) decreased the level of P-STAT3 and (**D**) increased the level of PPARα (*n* = 3) (200×, scale bar: 100 μm). The data are shown as the mean ± S.E.M. # *p* < 0.05, ### *p* < 0.001 vs. CTRL mice; * *p* < 0.05, ** *p* < 0.01, *** *p* < 0.001 vs. Alcohol mice.

IH enhanced the expression levels of 15-PGDH (*p* < 0.001) and PPARα (*p* < 0.01, Figure 5A,B) in the liver of chronic ALD mice, as screened by metabolomics analysis. Furthermore, IH suppressed the phosphorylation of STAT3 (*p* < 0.001) and JAK1 (*p* < 0.001) in the liver (Figure 5A,B). Based on IHC analysis, IH significantly reduced the expression of phospho- (P-) STAT3 (*p* < 0.001, Figure 5C) and enlarged the expression of PPARα (*p* < 0.001, Figure 5D) in the liver of chronic ALD mice.

## 4. Discussion

In this study, the effect of IH protecting against chronic ALD in mice via its property to relieve the inflammation response was systematically investigated. In chronic ALD mice, IH reversed steatosis and inflammatory cell infiltration in the liver, suppressed the hyper levels of ALT and AST in the serum, and increased the level of ADH in the liver. IH also regulated the levels of HDL-C, LDL-C, TG and TC in serum. These results partly suggested that IH ameliorated the chronic ALD and regulated the lipid metabolism. 

The rate of alcohol metabolism activity is suppressed in the liver of chronic ALD patients, which is directly regulated by ADH activity [28]. ADH can catalyze alcohol to acetaldehyde, and to some extent, the activity of ADH determines the rate of alcohol metabolism [27]. Previous studies have found that, although the expression of ADH is positively stimulated by ethanol content, long-term ethanol induction can lead to a decrease in ADH [28]. In addition, the decrease in alcohol metabolism rate increases the risk of lipid injuries [29]. IH treatment protected chronic ALD mice and improved ADH-related alcohol metabolism activity.

Intestinal microbiota is responsible for ALD [30]. In chronic ALD mice, IH increased the abundance of *Coprococcus*, *Bifidobacterium, Bifidobacteriales*, *Lactobacillus*, and *Phascolarctobacterium* associated with short-chain fatty acid (SCFA) production [31,32]. *Lactobacillus* is a prominent SCFA-producing intestinal commensal bacterium that encodes ADH, which promotes alcohol metabolism and alleviates alcoholic liver injury [33]. *Phascolarctobacterium*, an SCFA-producing intestinal microbe, is associated with the metabolic condition of the host [34]. Intestinal-derived SCFAs are mainly composed of acetate, propionate and butyrate, with the latter being the most important part [35]. Butyrate modulates the immune response of intestinal macrophages by inhibiting histone deacetylases and reducing the levels of pro-inflammatory cytokines such as IL-6 [36]. Furthermore, butyrate exerts anti-inflammatory effects by upregulating the level of peroxisome proliferator-activated receptor gamma (PPARγ) [37] and ameliorating alcoholic fatty liver disease [38]. Unanticipated changes in intestinal microbiota caused by IH may indeed have played a role in chronic ALD mice. To further investigate, we performed a combined analysis of intestinal microbiota and serum metabolites. It is worth mentioning that *Phascolarctobacterium* also shows a positive correlation with ALA [39]. ALA, an *n*-3 PUFA, shows various kinds of biological activities, one of which is anti-inflammatory [40]. *n*-3 PUFAs can protect the liver from chronic ALD by mediating various mechanisms, such as reducing new fat formation in adipose tissue, enhancing lipid metabolism, alleviating liver inflammation and promoting intestinal homeostasis [41]. 

Serum metabolites are affected by bioactive metabolites from the intestinal microbiota that enter the host’s blood circulation [42]. In our study, the significantly altered metabolites were associated with the immune system and signal transduction. Both 14-HDoHE and 8-HEPE, sourced from *n*-3 PUFAs [23,24], are the hydroxylates of docosahexaenoic acid (DHA) and eicosapentaenoic acid, which are synthesized from ALA and were influenced by IH in chronic ALD mice [43]. 14-HDoHE, the optimal substrate of 15-PGDH, can produce 14-oxoDHA, which inhibits pro-inflammatory cytokines including IL-1β, IL-6 and TNF-α [23]. IH reversed the levels of 14-HDoHE and 15-PGDH, which indirectly enhanced the anti-inflammation activity of 14-oxoDHA. PPARα is a key factor during the development of ALD, due to its close correlation with lipid metabolism and inflammation [44]. 8-HEPE activates the PPARα to regulate lipid dyslipidemia and inflammation by inhibiting STAT [11,45]. IH upregulated the PPARα and controlled lipid metabolism. 

Based on previous research, JAK1/STAT3 signaling inhibition can relieve the inflammation and ameliorate liver disease [8,46]. The activation of STAT3 is associated with the inhibition of PPARα, and IH reversed this effect [11]. JAK is a non-receptor-like tyrosine kinase and a family of signaling molecules that bridges the intracellular structures of type I and type II cytokine receptors. When a cytokine binds to an extracellular receptor, JAK is phosphorylated, followed by the activation of its downstream molecule STAT [47,48,49]. In a liver injury model, hepatocyte-specific STAT3 knockout mice show reduced liver inflammation [50]. In this study, the activation of JAK1 and STAT3 was significantly inhibited by IH, which may be responsible for reliving chronic ALD. Moreover, increased pro-inflammatory cytokines such as TNF-α, IL-1β and IL-6 exacerbate liver injury [51], a key feature of ALD [52]. IL-6 activates the phosphorylation of JAKs by binding to its receptor [53,54]. IL-1β stimulates TNF-α production and TNF-α induces IL-1β production [55]. IH strongly suppressed the expression of IL-1β, IL-6, and TNF-α in the liver of chronic ALD mice. Overall, IH can protect the liver against chronic alcohol accumulation by inhibiting JAK1/STAT3 signaling, to relieve the inflammatory response. 

This study had some limitations. First, we only confirmed the liver protection of IH against chronic alcohol injury, but failed to expound on its active materials. Second, the relationship among intestinal microbiota, metabolites, and target proteins is still unclear, and the detailed mechanism for alleviating chronic ALD by IH requires further investigation.

## 5. Conclusions

In this study, IH protected the liver against chronic alcohol injury in mice by relieving inflammation related to JAK1/STAT3 signaling. Through valid multifaceted data, this study provides a theoretical basis and support for the use of dietary therapy using IH to mitigate chronic alcoholic liver injury.

## Data Availability

Not applicable.

## Supplementary Materials

The following supporting information can be downloaded at: https://www.mdpi.com/article/10.3390/nu15163530/s1, Figure S1: Histopathological analysis of the organs (spleen, kidneys, heart, lungs, brain, and muscle) of mice using H&E staining (200×; scale bar: 100 μm) (*n* = 3); Figure S2: The proportion of identified metabolites in each chemical class; Table S1: Details of the factors used in detection kits; Table S2: Details of antibodies used in Western blot and IHC; Table S3: Effects of IH on organ indices in mice with alcohol-induced chronic liver injury; Table S4: Relative abundances of the top 20 genera in the three groups; Table S5: Significantly different serum metabolites among three groups.
