# Supplementary material for: Protection of Inonotus hispidus (Bull.) P. Karst. against Chronic Alcohol-Induced Liver Injury in Mice via Its Relieving Inflammation Response
Source: Nutrients. 2023 Aug 10;15(16):3530. doi: 10.3390/nu15163530 (PMC10458315; doi:10.3390/nu15163530)
Supplement: Supplementary file 1 [file nutrients-15-03530-s001.zip › nutrients-2529797-supplementary.pdf]

Supplementary Data

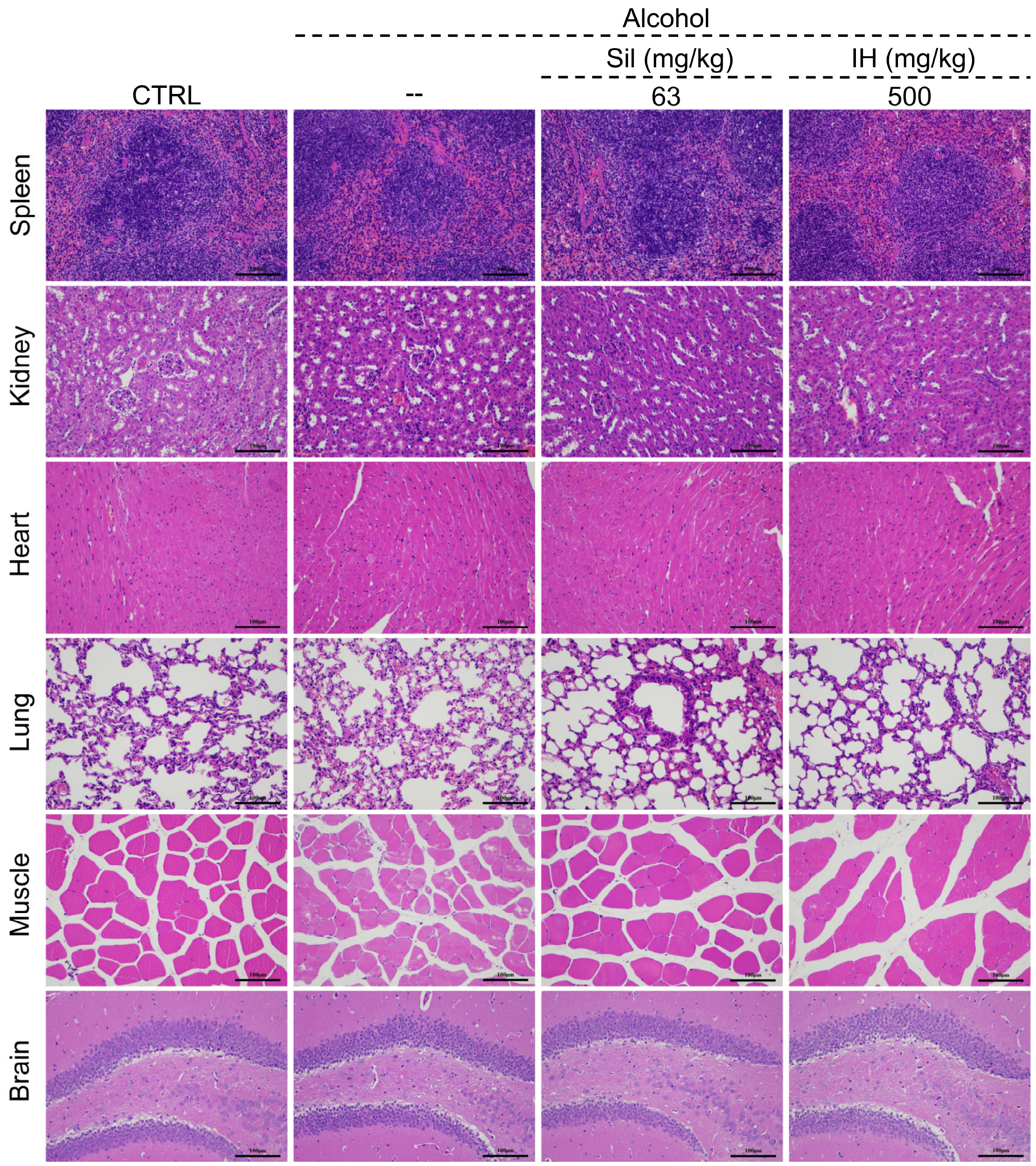

Figure S1. Histopathological analysis of the organs (spleen, kidneys, heart, lungs, brain, and muscle) of mice using H&E staining (200×; scale bar: 100 μm) (n = 3).

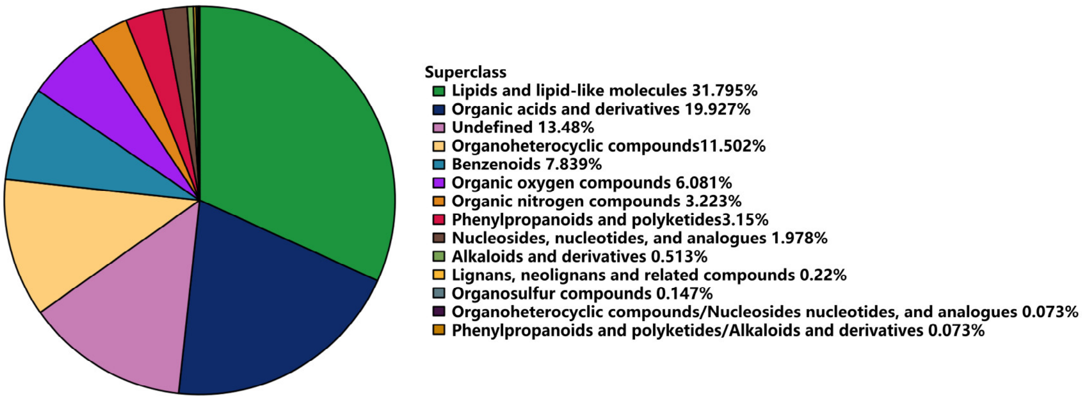

Figure S2. The proportion of identified metabolites in each chemical class.

Table S1. Details of the factors used in detection kits.

Factors	Catalog number	Company	Area
AST	C010-2-1	Nanjing Jiancheng Bioengineering Institute	Nanjing, China
ALT	C009-2-1	Nanjing Jiancheng Bioengineering Institute	Nanjing, China
TG	A110-1-1	Nanjing Jiancheng Bioengineering Institute	Nanjing, China
TC	A111-1-1	Nanjing Jiancheng Bioengineering Institute	Nanjing, China
LDL-C	A113-1-1	Nanjing Jiancheng Bioengineering Institute	Nanjing, China
HDL-C	A112-1-1	Nanjing Jiancheng Bioengineering Institute	Nanjing, China
ADH	G0874W	Suzhou Grace Biotechnology Co., Ltd	Suzhou, China
IL-1β	88-7013-88	Thermo Fisher Scientific	MA, USA
IL-6	88-7064-77	Thermo Fisher Scientific	MA, USA
TNF-α	88-7324-77	Thermo Fisher Scientific	MA, USA

Table S2. Details of antibodies used in Western blot and Immunohistochemistry (IHC).

Antibody	Molecular weight	Catalog number	Dilution for Western blot	Dilution for IHC	Company	Area
IL-1β	31 kDa	A16288	1:2000	/	Abclonal	Wuhan, China
IL-6	23 kDa	bs-0782R	1:2000	/	Bioss	Beijing, China
TNF-α	17/25 kDa	A0277	1:2000	/	Abclonal	Wuhan, China
PPARα	52 kDa	ab2779	1:500	1:400	Abcam	Cambridge, UK
P-STAT3	86 kDa	AF3293	1:2000	1:200	Affinity	Jiangsu, China
T-STAT3	92 kDa	3096489	1:2000	/	MILLIPORE	Shanghai, China
P-JAK1	130/133 kDa	AP0530	1:2000	/	Abclonal	Wuhan, China
T-JAK1	133 kDa	A5534	1:1000	/	Abclonal	Wuhan, China
15-PGDH	29 kDa	A5024	1:2000	/	Abclonal	Wuhan, China
β-actin	43 kDa	sc-47778	1:2000	/	Santa Cruz	TX, USA
Goat Anti-Rabbit	/	E-AB-1003	1:2000	1:400	Elabscience	Wuhan, China
Goat-Anti-Mouse	/	E-AB-1001	1:2000	1:400	Elabscience	Wuhan, China

Table S3. Effects of IH on organ indices in mice with alcohol-induced chronic liver injury.

	CTRL	Alcohol		
		--	Sil (63mg/kg)	IH (500mg/kg)
Heart	0.565±0.010	0.615±0.013$^{\#}$	0.562±0.012*	0.546±0.016**
Liver	3.749±0.042	5.019±0.063$^{\#\#\#}$	4.439±0.089***	4.764±0.105
Spleen	0.286±0.006	0.237±0.007$^{\#\#\#}$	0.256±0.005	0.244±0.008
Lung	0.537±0.015	0.658±0.024$^{\#\#\#}$	0.559±0.015***	0.583±0.008**
Kidney	1.210±0.018	1.425±0.019$^{\#\#\#}$	1.302±0.018***	1.314±0.020***
Thymus	0.121±0.007	0.093±0.007$^{\#}$	0.112±0.005	0.109±0.010

The data were analyzed using a one-way analysis of variance (ANOVA) and expressed as means ± S.E.M. $^{\#}P$ <0.05, $^{\#\#\#}P$ < 0.001 vs. CTRL mice; $^{*}P$ <0.05, $^{**}P$ <0.01, $^{***}P$ <0.001 vs. Alcohol mice.

Table S4. Relative abundances of the top 20 genera in the three groups.

Taxa	CTRL	Alcohol	Alcohol + IH
Allobaculum	0.198	0.063	0.068
Oscillospira	0.029	0.073	0.046
Coprococcus	0.012	0.036	0.057
Paraprevotella	0.009	0.038	0.011
Bacteroides	0.015	0.017	0.013
Desulfovibrio	0.021	0.020	0.004
Lactobacillus	0.016	0.008	0.010
Akkermansia	0.002	0.027	0.002
Ruminococcus	0.008	0.007	0.013
Clostridiaceae_Clostridium	0.005	0.006	0.012
Sutterella	0.008	0.008	0.004
Bifidobacterium	0.001	0.003	0.015
Phascolarctobacterium	0.005	0.000	0.012
Prevotella	0.002	0.004	0.009
Turicibacter	0.002	0.003	0.007
Parabacteroides	0.003	0.004	0.002
Alistipes	0.002	0.004	0.002
[Ruminococcus]	0.004	0.002	0.001
Flexispira	0.002	0.002	0.002
Rikenella	0.001	0.002	0.002

Data are presented as the mean.

Table S5. Significantly different serum metabolites among three groups.

Metabolites	CTRL	Alcohol	Alcohol + IH	CTRL vs Alcohol		Alcohol vs Alcohol + IH	
				VIP	*P*-value	VIP	*P*-value
14-hydroxy-4z,7z,10z,12e,16z,19z-docosahexaenoic acid	319302	152253	280111	2.535	0.006	2.937	0.010
8-hydroxy-5z,9e,11z,14z,17z-eicosapentaenoic acid	348249	190201	320936	2.432	0.004	2.894	0.018
2-dhahma [dmed-fahfa]	456700	697868	325016	3.553	0.001	3.380	0.024
N-acetyl-l-methionine	17419	38533	23013	1.115	0.001	1.071	0.024
2-oleoyl-1-stearoyl-sn-glycero-3-phosphoserine	183798	360380	252004	2.886	0.002	3.502	0.034
N-palmitoyl-d-erythro-dihydroceramide-1-phosphate	104406	152079	82078	1.495	0.003	2.713	0.014
L-dihydroorotate	1019771	694699	964423	4.276	0.004	4.637	0.009
3-amino-2-(4-chlorophenyl)-2-hydroxypropanesulfonic acid	62928	30797	53800	1.405	0.011	1.167	0.033
Pseudouridine	73606	50869	82612	1.025	0.029	1.647	0.013
Linolenic acid	1139523	741289	1115367	4.672	0.017	5.595	0.094

Data are presented as the mean. The metabolites were significantly different when variable important in projection (VIP) > 1 and *p*-value < 0.05.
